# SERUM METABOLOMICS SIGNATURES OF EXTREME OLD AGE AND LONGEVITY

**DOI:** 10.1093/geroni/igad104.2059

**Published:** 2023-12-21

**Authors:** Stefano Monti, Paola Sebastiani, Zeyuan Song, Ziwei Huang, Stacy Andersen, Thomas Perls

**Affiliations:** Boston University, Boston, Massachusetts, United States; Tufts Medical Center, Boston, Massachusetts, United States; Boston University, Boston, Massachusetts, United States; Physics Department, Boston University, Boston, Massachusetts, United States; Boston University Chobanian & Avedisian School of Medicine, Boston, Massachusetts, United States; Boston University, Boston, Massachusetts, United States

## Abstract

A total of 1,495 chemicals including 1,213 compounds of known identity and 282 compounds of unknown structural identity were profiled in serum samples collected at enrollment at Metabolon, Inc. from the blood serum of 213 subjects, including centenarians (n=80), offspring (n=70), and controls (n=63), mean ages of 105, 70, and 70, respectively, enrolled in the New England Centenarian Study (NECS). We performed metabolite- and metabolite module-based regression analyses on age, differential analyses comparing centenarian to offspring and control, and Cox proportional hazard-based survival analyses. We annotated the derived signatures by enrichment analysis using metabolite sets curated by Metabolon, RefMet, and Pathbank, among others. We identified 323 (436) metabolites that change with age at an FDR-corrected q-value of 0.01 (0.05), and 298 (407) metabolites differentially abundant between centenarians, their younger offspring, and controls. Our results confirm and expand upon previous metabolomics-based studies of aging, including decreased abundance with age of Tryptophan, DHEA-S, Pregnenolone, Glutathione, and Androgen, among others, and increased abundance of Amino acids (Tyrosines), Phenylacetylglutamine, Ornithine, Urea, Kynurenine, and Creatine, among others. To distinguish metabolites associated with longevity from those associated with extreme old age, we performed age-adjusted survival analysis, and rank-based enrichment analysis identified several metabolites significantly associated with survival independent of age, including secondary and primary (C 24) bile acids, and multiple classes of steroids (androgens, pregnenolone, progestin), among others. Additional analyses are ongoing to further annotate and characterize these findings. If validated, these results could contribute to the identification of novel healthy aging therapeutics.

